# Author Correction: Arabidopsis proteome and the mass spectral assay library

**DOI:** 10.1038/s41597-021-00852-8

**Published:** 2021-02-23

**Authors:** Huoming Zhang, Pei Liu, Tiannan Guo, Huayan Zhao, Dalila Bensaddek, Ruedi Aebersold, Liming Xiong

**Affiliations:** 1 King Abdullah University of Science and Technology, Core Labs, Thuwal, Kingdom of Saudi Arabia; 2 Division of Biological and Environmental Science and Engineering, King Abdullah University of Science and Technology, Thuwal, Kingdom of Saudi Arabia; 3 Department of Biology, Institute of Molecular Systems Biology, ETH Zurich, Zurich, Switzerland; 4 Faculry of Science, University of Zurich, Zurich, Switzerland; 5 Department of Biology, Hong Kong Baptist University, Kowlong Tong, Hong Kong, SAR China

**Keywords:** Proteomic analysis, Plant sciences, Proteomics, Arabidopsis thaliana

Correction to: *Scientific Data* 10.1038/s41597-019-0294-0, published online 22 November 2019

The spelling of King Abdullah University of Science and Technology has been corrected for author affiliations 1 and 2. This error has been corrected in both the HTML and PDF versions of this Data Descriptor.

